# ProteomicsDB: toward a FAIR open-source resource for life-science research

**DOI:** 10.1093/nar/gkab1026

**Published:** 2021-11-17

**Authors:** Ludwig Lautenbacher, Patroklos Samaras, Julian Muller, Andreas Grafberger, Marwin Shraideh, Johannes Rank, Simon T Fuchs, Tobias K Schmidt, Matthew The, Christian Dallago, Holger Wittges, Burkhard Rost, Helmut Krcmar, Bernhard Kuster, Mathias Wilhelm

**Affiliations:** Technical University of Munich, Computational Mass Spectrometry, 85354 Freising, Bavaria, Germany; Technical University of Munich, Chair of Proteomics and Bioanalytics, 85354 Freising, Bavaria, Germany; Technical University of Munich, Chair of Proteomics and Bioanalytics, 85354 Freising, Bavaria, Germany; Technical University of Munich, Chair of Proteomics and Bioanalytics, 85354 Freising, Bavaria, Germany; Technical University of Munich, Chair for Information Systems, 85748 Garching, Bavaria, Germany; Technical University of Munich, SAP University Competence Center, 85748 Garching, Bavaria, Germany; Technical University of Munich, Chair for Information Systems, 85748 Garching, Bavaria, Germany; Technical University of Munich, SAP University Competence Center, 85748 Garching, Bavaria, Germany; Technical University of Munich, Chair for Information Systems, 85748 Garching, Bavaria, Germany; Technical University of Munich, SAP University Competence Center, 85748 Garching, Bavaria, Germany; Technical University of Munich, Chair of Proteomics and Bioanalytics, 85354 Freising, Bavaria, Germany; Technical University of Munich, Chair of Proteomics and Bioanalytics, 85354 Freising, Bavaria, Germany; Technical University of Munich, Department for Bioinformatics and Computational Biology, 85748 Garching, Bavaria, Germany; Technical University of Munich, Center of Doctoral Studies in Informatics and its Applications (CeDoSIA), 85748 Garching, Bavaria, Germany; Technical University of Munich, Chair for Information Systems, 85748 Garching, Bavaria, Germany; Technical University of Munich, SAP University Competence Center, 85748 Garching, Bavaria, Germany; Technical University of Munich, Department for Bioinformatics and Computational Biology, 85748 Garching, Bavaria, Germany; Technical University of Munich, Institute for Advanced Study (TUM-IAS), 85748 Freising, Bavaria, Germany; Technical University of Munich, Chair for Information Systems, 85748 Garching, Bavaria, Germany; Technical University of Munich, SAP University Competence Center, 85748 Garching, Bavaria, Germany; Technical University of Munich, Chair of Proteomics and Bioanalytics, 85354 Freising, Bavaria, Germany; Technical University of Munich, Bavarian Biomolecular Mass Spectrometry Center (BayBioMS), 85354 Freising, Bavaria, Germany; Technical University of Munich, Computational Mass Spectrometry, 85354 Freising, Bavaria, Germany

## Abstract

ProteomicsDB (https://www.ProteomicsDB.org) is a multi-omics and multi-organism resource for life science research. In this update, we present our efforts to continuously develop and expand ProteomicsDB. The major focus over the last two years was improving the findability, accessibility, interoperability and reusability (FAIR) of the data as well as its implementation. For this purpose, we release a new application programming interface (API) that provides systematic access to essentially all data in ProteomicsDB. Second, we release a new open-source user interface (UI) and show the advantages the scientific community gains from such software. With the new interface, two new visualizations of protein primary, secondary and tertiary structure as well an updated spectrum viewer were added. Furthermore, we integrated ProteomicsDB with our deep-neural-network Prosit that can predict the fragmentation characteristics and retention time of peptides. The result is an automatic processing pipeline that can be used to reevaluate database search engine results stored in ProteomicsDB. In addition, we extended the data content with experiments investigating different human biology as well as a newly supported organism.

## INTRODUCTION

ProteomicsDB (https://www.ProteomicsDB.org) has developed into a multi-omics and multi-organism resource for life science research (1). It is built upon the in-memory-database technology HANA (2) enabling fast access to stored data and thus offering real-time data analytics capabilities. ProteomicsDB was originally developed to investigate large quantities of human quantitative mass spectrometry-based proteomics data, highlighted on one of the first drafts of the human proteome (3,4). However, over the past years it was extended to include additional organisms including *Mus musculus* and *Arabidopsis thaliana* (5) as well as additional omics types, such as transcriptomics and phenomics data (1,4). Because of this, ProteomicsDB has become a rich and valuable resource for life science research and extends beyond the scope of proteomics experiments. This is visible by the external resources integrating with ProteomicsDB, such as GeneCards (6), UniProt (7), OmniPathDB (8) and Gene Information eXtension (GIX) (9). Today, we notice on average ∼500 unique visitors per day.

A unique characteristic of ProteomicsDB is its ability to integrate large amounts of diverse data.

For example, while Expression Atlas (10) provides differential and baseline proteomics and transcriptomics data for a diverse set of organisms that can be explored online, the analysis is limited to the investigation of single experiments. In ProteomicsDB, the expression information across hundreds or thousands of experiments can be investigated simultaneously. In MaxQB (11), researchers are able to retrieve data from individual proteins similar to ProteomicsDB. However, the stored data are limited to proteomics with a limited number of distinct experiments. For example, the expression information of epidermal growth factor receptor (EGFR) in MaxQB covers 11 cell lines while ProteomicsDB provides information for 41 tissues and body-fluids as well as for 60 cell lines. For 52 of these, ProteomicsDB also provides cell viability information.

Large data stewards, like ProteomicsDB, have the obligation to provide access to its data content in a way that also enables other researchers to reproduce, reanalyze and integrate the data. The specific requirements and principles behind this concern the Findability, Accessibility, Interoperability, and Reusability (FAIR) of (research) data (12). Following this movement, additional work expanded these principles in order to account for (research) software as well (13). The need for this separation becomes clear when considering one concrete principle. The reusability aspect of data is met when rich descriptions of the data are made available in a common data format. For software, this principle is additionally linked to the maintainability of the codebase. This includes the availability of appropriate documentation of the source code (13). The FAIR principles are at the very core of open science and are essential for the scientific community to use generated data effectively. As such, they were a major focus guiding the development of ProteomicsDB over the last 2 years.

In this update, we discuss the developments of ProteomicsDB of the last two years, and specifically highlight our progress in turning ProteomicsDB into a FAIR and open source resource for life science research. For that purpose, we designed and implemented a reference architecture for ProteomicsDB (14) to enable fast development of new services and keep these services maintainable, manageable and extendable in future. Based on this, we created a new API that gives users access to essentially all data stored in ProteomicsDB achieving a major step toward enabling FAIR data access. We also release an open-source re-implementation of the user interface (UI) that not only turns the frontend into a reusable and expandable resource by external developers but also brings ProteomicsDB in accordance with modern web standards. In light of this, a new visualization was added that shows the primary, secondary and tertiary structure of proteins. In addition, we imported new data into ProteomicsDB, including data from a new organism, rice (*Oryza sativa* ssp. *japonica*), and we created a pipeline to improve the quality of the proteomics data stored within ProteomicsDB by using Prosit, a deep neural network that can predict various properties of peptides (15,16).

## RESULTS

### Full access to data stored in ProteomicsDB via new API

**Figure 1.**
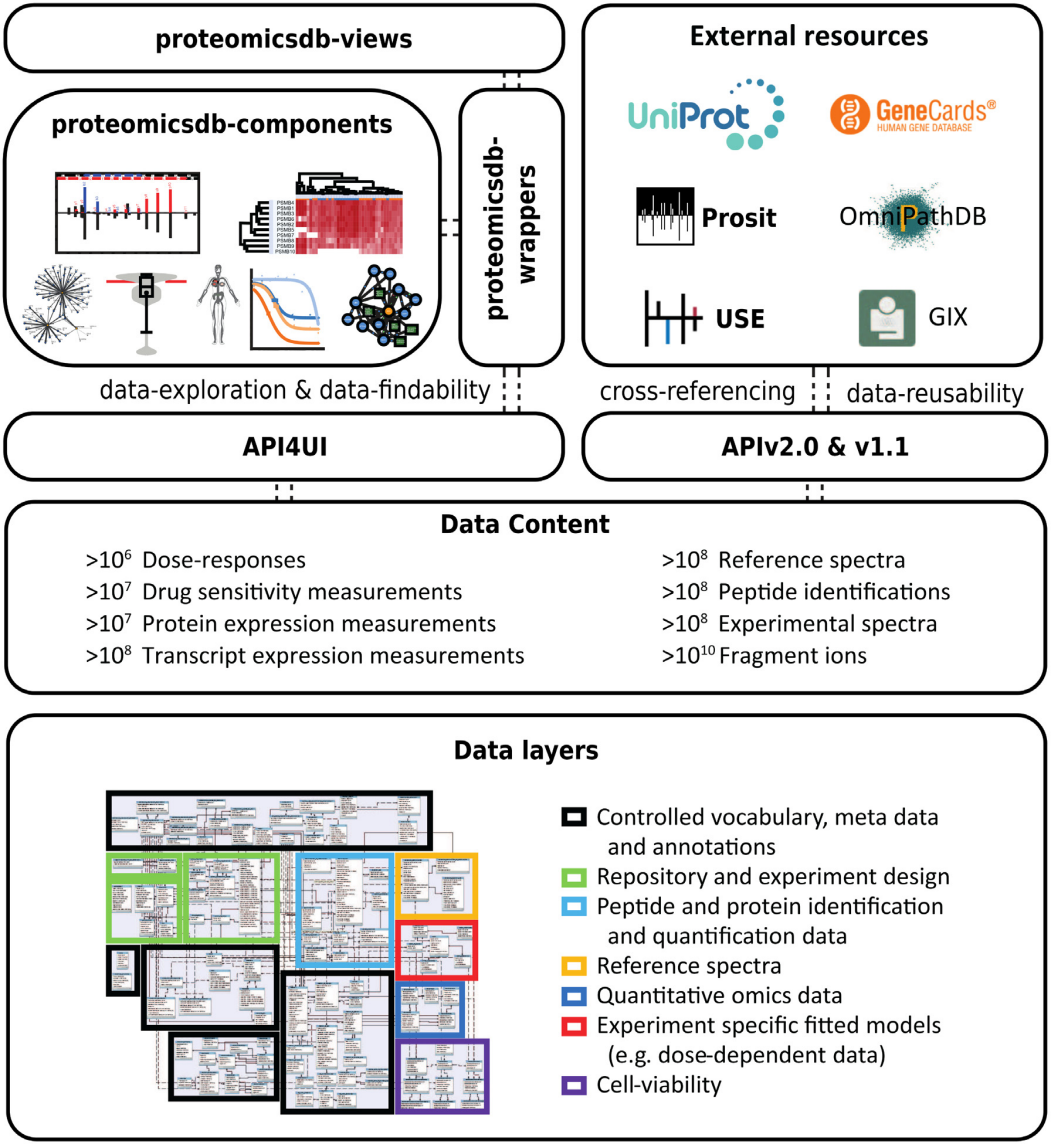
The architecture of ProteomicsDB. The data content and data layer of ProteomicsDB are accessible via three application programming interfaces (APIs). The API4UI is used by the frontend and contains predefined requests to the data in ProteomicsDB for the purpose of data visualization. The novel vue-based visualization layer of ProteomicsDB (top left) is separated into three levels. The proteomicsdb-components package is agnostic toward ProteomicsDB and thus usable on any website. The package proteomicsdb-wrappers connects the components with ProteomicsDB and can be re-used on any website as well. The package proteomicsdb-view contains the entire vue-based frontend of ProteomicsDB. The APIv1.1 is used by external resources (top right) and will remain publicly available. The new APIv2.0 provides access to virtually any datasets stored in ProteomicsDB.

ProteomicsDB offered access to its data in form of an application programming interface (API) since its inception. However, the available APIs limited access to 10 predefined views all centered on the proteomics data. Already then, users did not have access to a large number of internal tables storing information on, for example, the used controlled vocabularies and neither to the newly supported omics data added in the past years. For this reason, we developed a new central API version two here (APIv2.0) that provides access to essentially all data currently stored in ProteomicsDB (Figure 1). During its development, we followed the guidelines and recommendations of the FAIR principles (12) with a focus to make the API of ProteomicsDB accessible and usable for both (non-)bioinformatics researchers and developers. The new version incorporates the functionality of all previously offered APIs turning it into the central (programmatic) access point to the data stored in ProteomicsDB.

An important aspect of offering FAIR data access is to use established standards. For this reason, we decided to use the OData Version 2.0 Protocol (https://www.odata.org/documentation/odata-version-2-0/overview/). OData is used for creating HTTP-based data services that can be queried by web clients using standard HTTP messages and respond in a standardized structure. For each OData service, metadata concerning the service is automatically created. This ensures the compliance of all already created and all future API endpoints regarding their findability, accessibility, interoperability and reusability. Furthermore, OData offers a large set of automatically generated functionalities, such as filtering and data formatting [in JSON (17) and XML (18)]. These features are consequently all available in our APIv2.0.

**Figure 2.**
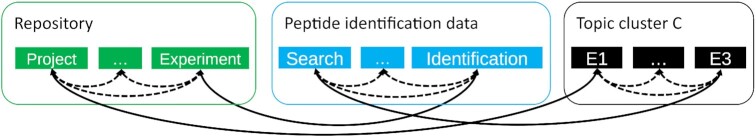
APIv2.0. The tables of ProteomicsDB are grouped into topic clusters (e.g. Repository and Peptide identification data, see Figure 1 data layers). Each table is available in the API as a separate entity (square boxes). To navigate between entities with (dashed black arrows) or across (solid black arrows) topic clusters, corresponding navigation properties were defined that allow the traversal of the available data. A detailed documentation of the API is available online under https://www.proteomicsdb.org/vue/apiv2/.

For easier navigation, we separated the entire data model of ProteomicsDB into 19 topic clusters. A topic cluster groups multiple entities (e.g. samples and experiments) that contain information about a similar content type (e.g. the repository or transcriptomics data). For example, the repository of ProteomicsDB is such a topic cluster (Figure 2) where the data and relation between projects, experiments, samples, files, measurements and supplementary files can be queried. The APIv2.0 allows to query in total 93 entities. To query an entity, the URL only contains the requested entity, e.g. /api_v2/api.xsodata/Sample. This query will return the descriptions and metadata to all available samples in ProteomicsDB.

A central objective of the APIv2.0 was that users can navigate from one entity to another. This was realized by the ‘navigation properties’. These navigation properties allow users an easy traversal between entities in multiple directions. For example, from the list of samples users can navigate to a list of all files that are connected to this sample or navigate to the respective experiment of that sample (Figure 2). This can be achieved by querying for ‘/api_v2/api.xsodata/Sample(ID)/File’ or ‘/api_v2/api.xsodata/Sample(ID)/Experiment’, respectively. This feature is available for all entities within a topic cluster and where possible across topic clusters. With this step, we simplify access and allow users to systematically query for data originally separated into multiple APIs. In accordance with the FAIR principles, all entities in ProteomicsDB come with a Global Unique Identifier (G_UID) that follow the format: *PRDB_UID:PRDB:<EntityName>:<LoacalIDOfEntity >* .

A detailed description of the APIv2.0 is available online (https://www.proteomicsdb.org/vue/apiv2/). Here, we list all available entities, their attributes (columns) and possible navigation properties to other entities. Additionally, each navigation property and entity listed also includes an example request. In order to find relevant entities and navigation properties, we implemented a search functionality that allows searching for any content listed in the API documentation (i.e. entities, attributes, navigation properties and examples).

We are continuously working on extending ProteomicsDB and due to this, the APIv2.0 will also be subject to changes, such as the addition of new navigation properties, entities and columns. The newly developed reference architecture for ProteomicsDB (14) enables versioning. Because of that, currently available endpoints will remain available even in the rare event of modifications to the internal representation of the data. When using the APIv2.0, we recommend to only request necessary data by using e.g. the filtering options of OData to reduce the overall response time as the largest table of ProteomicsDB exceeds 40 billion entries. The new API is a substantial improvement over the status quo and will enable scientist to benefit from the wealth of data stored in ProteomicsDB as well as an easier integration of data from ProteomicsDB into their applications and databases.

### Open-source ProteomicsDB frontend via reimplementation in Vue.JS

The current user interface (UI) of ProteomicsDB was built based on a SAP specific framework, termed SAPUI5. However, even its open-source variant, OpenUI5, is infrequently used in research. Due to this, developers in the field of life science research are unlikely to integrate or reuse the applications and visualizations developed for ProteomicsDB. Hence, open-sourcing the current UI is of little value to the scientific community. In accordance with our goal of turning ProteomicsDB into a FAIR resource, we set out to re-implement the UI of ProteomicsDB focusing on modularity, reusability and flexibility. The current version of the re-implementation (https://www.proteomicsdb.org/vue) covers all functionality required to browse and interact with the results stored for a single protein of interest as well as two analytics.

We selected Vue.js (https://vuejs.org/) in combination with the Vuetify (https://vuetifyjs.com/en/) package as the new frontend framework. This decision was made because of two reason. First, it is intuitive and well documented, which is important for creating a maintainable and reusable UI. Especially (external) developers interested in generating a new visualization will benefit from this. Second, the component system (modularization) of Vue.js allows easy encapsulation of functionality and subsequently reuse of visualizations. In line with our goal to improve the FAIRness of ProteomicsDB, we decided to exploit this core feature of Vue.js and separate our new interface into three functional levels (Figure 1, top left). The package proteomicsdb-components (https://github.com/wilhelm-lab/proteomicsdb-components) provides the base functionality for different visualization used in ProteomicsDB. They are agnostic to ProteomicsDB and thus can be reused on any website without specific dependencies and can be connected to any other source of data. The package proteomicsdb-wrappers provides wrappers (https://github.com/wilhelm-lab/proteomicsdb-wrappers) for these visualizations that request the data from ProteomicsDB. These wrappers can also be used on any website but will require a connection to ProteomicsDB. Last, these visualizations are combined into views in the package proteomicsdb-views (https://github.com/wilhelm-lab/proteomicsdb-views) that can be thought of as subpages in ProteomicsDB.

All of these levels are publicly available on GitHub as separate repositories. We expect that this will further improvement the findability and accessibility, but particularly the reusability of the code base of ProteomicsDB. Each of the three repositories are identified by individual Digital Object Identifiers (DOI), while each version can be uniquely identified with the associated git commit hash.

**Figure 3.**
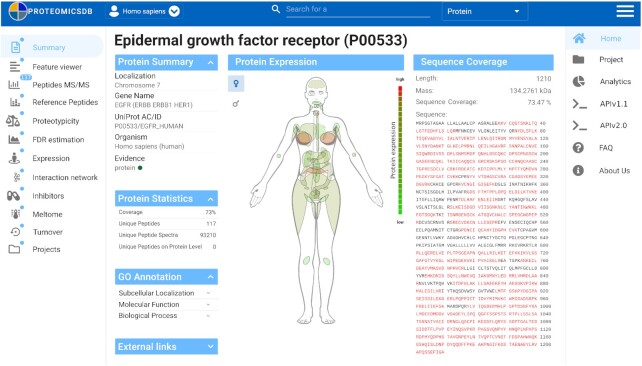
Screenshot of the new vue-based protein summary page. The organism selection is located at the top left next to the ProteomicsDB logo. In the top middle, a new universal search field was added visible at all times. The hamburger button on the top right opens the main navigation panel of ProteomicsDB. On the left, the protein navigation panel is shown. The protein summary page shows general information about the selected protein as well as the sequence coverage and the expression of the protein for tissues and body fluids.

With the switch to Vue.js and the reimplementation necessary for that, we also decided to redesign the layout of ProteomicsDB to provide a more intuitive and modern looking experience (Figure 3). The organism selection previously located on the left of the screen is now moved to a drop-down menu located at the top left, next to the ProteomicsDB logo. The main tabs that were previously at the top of the screen can now be access on the right side of the screen after clicking the three stacked horizontal bars (hamburger button) in the top right of the screen. Otherwise, they are hidden to dedicate a larger proportion of the screen to the current view. At the top center of the screen a new universal search field can be found that can be used as direct entry point to all aspects of ProteomicsDB.

After searching for a gene of interest and selecting a specific protein/isoform, the UI changes and a second menu appears on the left. This menu shows the different navigation options to investigate, for example, the observed peptides or expression pattern. The blue bubbles indicate whether and how much data are available in this view, for example, 137 distinct peptides identified for protein EGFR (Figure 3). The views available here are largely identical to the old UI, but some slight adjustments were made. For example, the biochemical assay tab was split into three separate views that show the available binding data for different inhibitors, melting behavior and turnover data.

**Figure 4.**
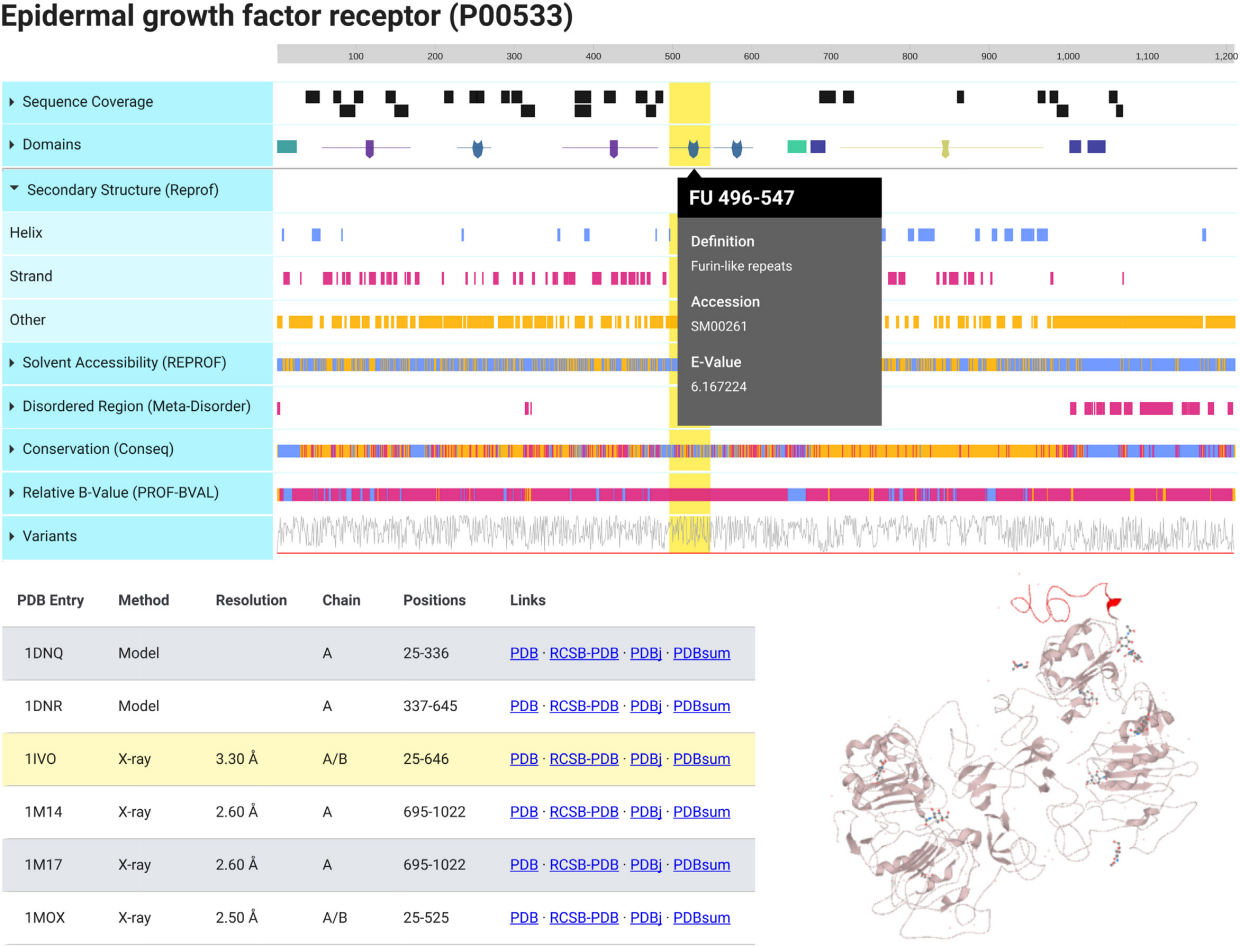
Protein *Feature Viewer*. This interactive visualization depicts different information about the primary and secondary structure about the protein in separate tracks. Each of these tracks can be expanded to reveal a more detailed view, exemplified by the expanded predicted secondary structure. Each region of a track can be selected to reveal additional information, exemplified for the Furin-like-repeats domain. In the bottom left, the table shows available 3D structures from PDB for this proteins. The selected structure is shown in the bottom right and the selected region (yellow highlight) is marked in red in the protein structure.

In addition to the redesign of the UI, two new visualizations were created for ProteomicsDB. First, the *Feature Viewer* (Figure 4), which is a custom adjustment (https://github.com/wilhelm-lab/protvista-proteomicsdb) of protvista-uniprot (https://github.com/ebi-webcomponents/protvista-uniprot) that depicts primary (e.g. sequence coverage and conservation) and secondary (e.g. domains, solvent accessibility and disordered regions) structure information of the selected protein. The properties shown originate from internal data or external resources (19–22) and are shown as separate tracks. Each track can be expanded to reveal a more detailed view (Figure 4, secondary structure), while a specific region of one attribute can be selected to reveal additional information (Figure 4, gray popup on the domain FU 496–547). In addition, available 3D structures are retrieved from PDB (22) and listed. A single structure can be selected (Figure 4, bottom left table, yellow highlight) and interactively investigated (Figure 4, bottom right structure viewer). If present in the structure, regions selected in the attribute view are automatically highlighted in the structure (Figure 4, yellow region highlighted in red in the 3D structure).

**Figure 5.**
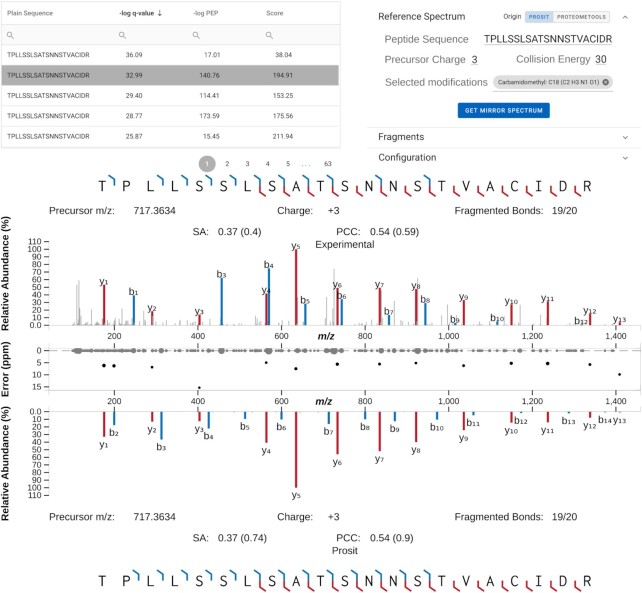
*Spectrum viewer*. The spectrum viewer (bottom) visualizes the selected peptide spectrum match from the table in the top left. The configuration element on the top right can be used for, but is not limited to, retrieving reference spectra depicted in the mirror view to the bottom. Reference spectra can be generated in real-time by Prosit or requested from ProteomeTools. In between the experimental and reference spectrum, the alignment error between an observed and reference peak is shown in parts-per-million (ppm). The spectral similarity between the experimental and reference spectrum is measured by calculating the Pearson correlation (PCC) and normalized spectral contrast angle (SA). The measures inside the brackets show the result of this comparison when taking either the peaks of the experimental or reference spectrum into account whereas the values outside the brackets show the measures calculated taking all peaks form both spectra into account.

The second example of a vastly improved visualization is the spectrum viewer (Figure 5) that is a modified version of the Universal Spectrum Explorer (23). It is accessible by selecting a specific peptide of interest in either the *Peptide MS/MS* or *Reference Peptides* view that show a table with the observed or synthetic/predicted reference peptides for the selected protein. As in the old version, every peptide spectrum match (PSM) stored in ProteomicsDB can be investigated here. Selecting a PSM (Figure 5, top left) fetches the associated spectrum. By default, a corresponding predicted reference spectrum is generated in real-time by Prosit and can be used to manually verify the correctness of the identification. In addition, reference spectra stored in ProteomicsDB from e.g. ProteomeTools (24) can be selected.

The reimplementation of the UI in Vue.js not only will enable external developers to be able to reuse views and visualization developed for ProteomicsDB but also shows that external views can be reused in ProteomicsDB. The availability of the source code on GitHub also creates a communication channel with users and developers that can report bugs and request new features, all supporting the FAIRification of ProteomicsDB.

### Increasing peptide and protein coverage by rescoring of FAIR data

Our recently described deep-neural-network Prosit was trained to predict the fragment intensities and retention times of peptides (15). Such prediction can be used to improve the separation between correct and incorrect matches of database search engine results (25). To achieve this, theoretical spectra of the proposed peptide sequences are predicted using Prosit and compared to the experimentally observed spectrum. Based on this, a variety of intensity-based scores are calculated. This rescoring process supports that published datasets often contain more information than what was initially discovered (26) and that FAIR datasets are a rich resource for novel findings. Additionally, it can be used to align and compare the results obtained from different database search engines (16).

**Figure 6.**
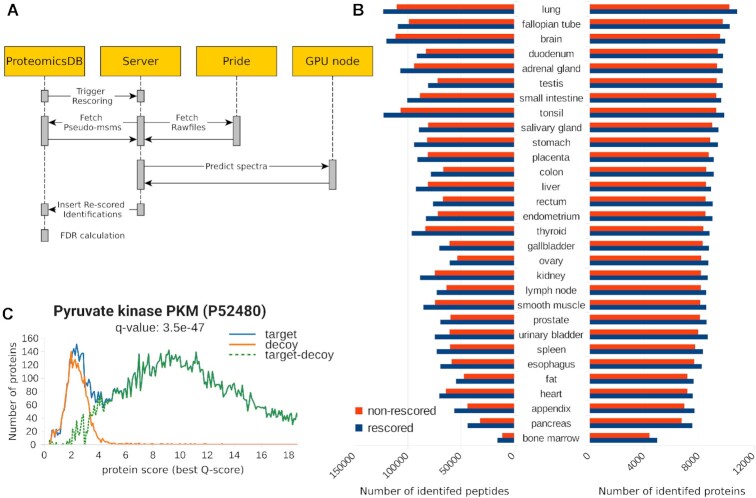
Integration of Prosit into ProteomicsDB. (**A**) Depiction of the workflow implemented to enable automatic rescoring of projects in ProteomicsDB. Raw mass spectrometry data are downloaded from PRIDE. The rescoring is performed on the database search results stored in ProteomicsDB by retrieving predictions from Prosit. The resulting scores are merged by percolator and imported into ProteomicsDB where the picked protein approach is used for FDR estimation. (**B**) The number of proteins (right) and peptides (left) identified with (blue) and without (red) rescoring at an estimated PSM, peptide and protein FDR of 1% for 30 tissues from Wang *et al.* (29). (**C**) Distribution of target and decoy *Q*-scores of proteins supported by peptide identifications for all mouse proteins in ProteomicsDB. The example highlights the *q*-value of the Pyruvate kinase PKM (P52480).

Considering the large amounts of data made available via ProteomicsDB, we decided to integrate the rescoring workflow directly into ProteomicsDB to enable the automatic re-processing of any FAIR dataset. The workflow (Figure 6A) can be triggered on datasets which have an associated ProteomeXchange (27) identifier. The associated raw mass spectrometry files are then automatically downloaded from PRIDE (28). Together with the reconstructed database search engine results from ProteomicsDB, a regular rescoring by Prosit is triggered. Then the percolator results are imported into ProteomicsDB again. This does not overwrite any data of the original search results and during false discovery rate (FDR) estimation either the original search engines scores or the intensity-based scores from Prosit are used.

As a proof of principle, we rescored 30 tissues of the data published by Wang *et al.* (29) in which the proteomes and transcriptomes of healthy human tissues were characterized. When analyzing each tissue separately, on average 8289 (±1126 standard deviation, SD) proteins were identified without rescoring (Figure 6B). The rescoring approach identified on average 8788 (±1088 SD) proteins across the different tissues. This is equal to an average relative increase of 6%. The largest benefit we observed was for bone marrow with a relative increase of 13%. The data for the small intestine benefited least from the rescoring but still showed an increase in the number of identified proteins by ∼4%. The effect on peptide level was even more pronounced. The number of identified peptides increased on average by 16% from 71 631 (±22 216 SD) to 82 165 (±22 209 SD). The tissues which benefited the strongest and the least on peptide level were bone marrow and brain with an increase of 40% and 7%, respectively. The large effects seen in bone marrow on peptide and protein level are most likely due to the overall lower number of identifications in this tissue. The biggest relative effect was observed for tissues with the smallest number of identified peptides without rescoring. This is consistent with previous observations that the rescoring is most beneficially when the identification rate is unexpectedly low, likely due to a strong overlap in targets and decoy matches (15).

In order to safely allow the combination of rescored and non-rescored data, we modified the FDR estimation procedure implemented in ProteomicsDB. As described earlier (30), we utilize *Q*-scores (-log_10_*q*-values) in order to combine results from different result sets. Figure 6C shows the *Q*-score distribution of target and decoy proteins. Here, the mouse data were chosen because of its high ratio of rescored data. The high degree of overlap between the number of estimated false positives (decoys) and likely incorrect targets in the low scoring region suggests that no bias is visible for proteins being supported by either rescored data or non-rescored data. This is further supported by the estimated distribution of true positives (target-decoy) that does not show any bimodality, suggesting that the decoy distribution accurately resembles the distribution of false matches in the target database.

The systematic rescoring of datasets in ProteomicsDB is only possible due to resources such as PRIDE which enable the findability, accessibility, interoperability and reusability of raw mass spectrometry files. With the full integration of the rescoring approach into ProteomicsDB, the number of peptides and confidence in their identification can be increased. With the ever growing amount of data available in ProteomicsDB, accurately assessing the confidence of peptide spectrum matches will remain a challenge which will require regular checks to be able to assure high overall data quality.

### Increasing the findability of aggregated data by ProteomicsDB

**Figure 7.**
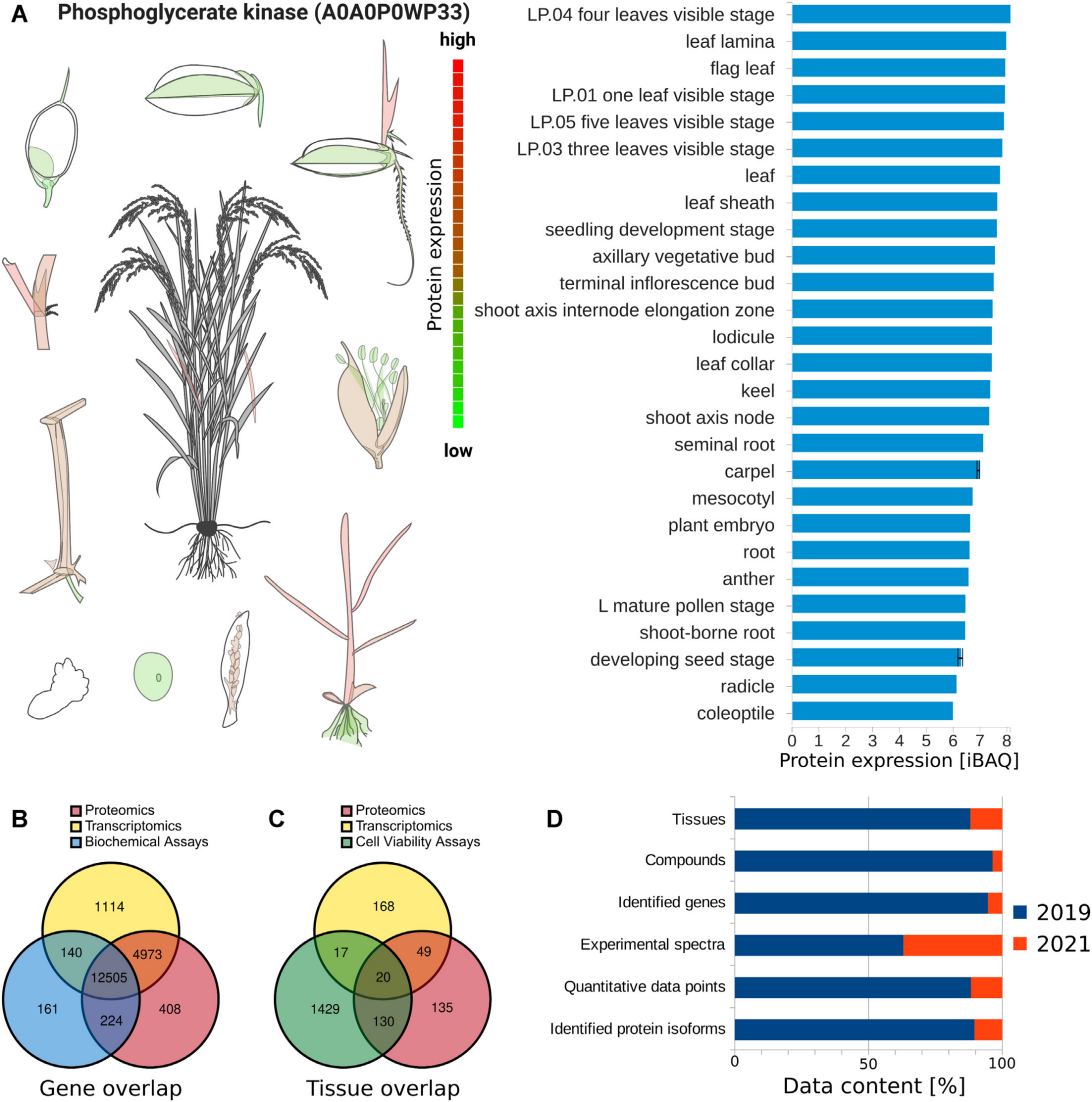
New data added to ProteomicsDB. (**A**) Expression bodymap (left) of rice illustrated on the example for Phosphoglycerate kinase (A0A0P0WP33). The individual expression values are depicted in the barplot (right). (**B**) Venn diagram showing the overlap of human genes, for which proteomics, transcriptomics or biochemical assay data is available in ProteomicsDB. (**C**) Venn diagram showing the overlap of human tissues, cell lines and body fluids for which proteomics, transcriptomics or cell viability assay data are available in ProteomicsDB. (**D**) Barplot showing the increase in data across the depicted categories (*y*-axis) from 2019 to 2021.

ProteomicsDB is the central point of access to aggregated information (e.g. protein expression) for a majority of its stored datasets and by that fosters their FAIRness. Over the last 2 years, many additional datasets were added to ProteomicsDB (Figure 7). We imported proteomics data from 32 projects investigating different human biology (29,31–65) that represent data on 40 new tissues and cell lines. In total, over 57 million experimental spectra and >500 thousand quantitative data points were added to ProteomicsDB. Considering the large amount of data previously available in ProteomicsDB, the effect on the number of identified proteins and genes is not less substantial, raising the confidence of 1281 protein isoforms and 878 genes to meet the <1% FDR criteria.

Especially the FAIRness of dataset reporting aggregated data beyond protein expression values (e.g. melting curves or dose response curves) benefit from ProteomicsDB because even fewer resources exist for those. Most often such data are only available in the supplement of the original publication hampering FAIRness. Recently, we added protein-drug binding data, covering a new class of proteins, histone deacetylases (HDACs). The inhibition of HDACs has shown promise as therapeutic option in oncology and other conditions such as Duchenne Muscular Dystrophy (66). We imported data for 53 HDAC inhibitors covering 14 target proteins, totaling 735 HDAC dose–response curves (67).

Most notably, we extended ProteomicsDB to support the storage and visualization of data for a new organism, *Oryza sativa* ssp. *Japonica* (rice) (Figure 7A). All functionalities of ProteomicsDB readily transfer to new organisms. For example, the visualization of expression values on a ‘bodymap’ (Figure 7A) only require the addition of a new organism visualization while the data retrieval, mapping and coloring of tissues is implemented generically. The imported data covers 28 rice tissues. In total, >4 million experimental spectra were imported resulting in the confident identification of close to 170 thousand distinct peptides of which >150 thousand are unique on gene level. Due to the imported data, 2621 of the 4051 annotated rice genes are confidently identified resulting in a coverage of 64%. For proteins isoforms, 13 742 of the 43 671 annotated were identified, resulting in an isoform coverage of 31%.

## FUTURE DIRECTIONS

The updates introduced over the last two years provide a solid foundation of turning ProteomicsDB into a FAIR resource for life science research. There are three specific objectives we aimed to support by this. First, foster data re-use for wet- and dry-lab researchers and allow them to utilize and benefit from the wealth of data available. Second, share our efforts in developing modern and easy-to-use web applications. Third, switch the development of ProteomicsDB to a community-driven effort. For this purpose, we are also currently developing a community portal within ProteomicsDB to allow users to share and discuss ideas about new visualization and features. At the time of writing, a direct line of communication between users and the current developers was established via GitHub where users can report discovered bugs or request new features. Ultimately, the availability of a comprehensive API and open source UI may lead to external developers contributing novel tools and analytics to ProteomicsDB.

The integration of Prosit into ProteomicsDB enables the rescoring of all data stored in ProteomicsDB. On individual datasets, we observed an average increase in the number identified peptides by 16% and proteins by 6%. When performed on all data, this may increase the coverage of ProteomicsDB substantially and increase the quantitative precision by increasing the number observed peptides used to quantify each protein. In addition, this allows us to combine multiple database search engine results across and within datasets and will eventually enable us to integrate the results of novel search engines.

A strong focus of the next years will be on the finalization of the new interface, as well as the integration of substantially more data. Particularly the extension to support the storage, visualization and integration of data from experiments that investigated post-translational modifications will be of high priority. For this, new views and visualization are required, which can be developed much faster by the migration to the new reference architecture and Vue.js. We expect that the publicly available API and open source implementation of the UI will facilitate the development of novel applications and analytics. We further envisage that ProteomicsDB can be made available as private instances for research institutions, consortia or individual labs.

## DATA AVAILABILITY

ProteomicsDB is available at https://www.ProteomicsDB.org. Protvist-proteomicsdb is available at https://github.com/wilhelm-lab/protvista-proteomicsdb. Proteomicsdb-wrappers is available at https://github.com/wilhelm-lab/proteomicsdb-wrappers. Proteomicsdb-components is available at https://github.com/wilhelm-lab/proteomicsdb-components. Proteomicsdb-views is available at https://github.com/wilhelm-lab/proteomicsdb-views.

## References

[1] Samaras P. , Schmidt T., Frejno M., Gessulat S., Reinecke M., Jarzab A., Zecha J., Mergner J., Giansanti P., Ehrlich H.-C. et al. ProteomicsDB: a multi-omics and multi-organism resource for life science research. Nucleic Acids Res. 2020; 48:D1153–D1163. 31665479 10.1093/nar/gkz974 PMC7145565

[2] Färber F. , May N., Lehner W., Große P., Müller I., Rauhe H., Dees J. The SAP HANA Database–An architecture overview. IEEE Data Eng. Bull. 2012; 35:28–33.

[3] Wilhelm M. , Schlegl J., Hahne H., Gholami A.M., Lieberenz M., Savitski M.M., Ziegler E., Butzmann L., Gessulat S., Marx H. et al. Mass-spectrometry-based draft of the human proteome. Nature. 2014; 509:582–587. 24870543 10.1038/nature13319

[4] Schmidt T. , Samaras P., Frejno M., Gessulat S., Barnert M., Kienegger H., Krcmar H., Schlegl J., Ehrlich H.-C., Aiche S. et al. ProteomicsDB. Nucleic Acids Res. 2018; 46:D1271–D1281. 29106664 10.1093/nar/gkx1029 PMC5753189

[5] Mergner J. , Frejno M., List M., Papacek M., Chen X., Chaudhary A., Samaras P., Richter S., Shikata H., Messerer M. et al. Mass-spectrometry-based draft of the Arabidopsis proteome. Nature. 2020; 579:409–414. 32188942 10.1038/s41586-020-2094-2

[6] Stelzer G. , Rosen N., Plaschkes I., Zimmerman S., Twik M., Fishilevich S., Stein T.I., Nudel R., Lieder I., Mazor Y. et al. The GeneCards Suite: from gene data mining to disease genome sequence analyses. Curr. Prot. Bioinform. 2016; 54:1.30.1–1.30.33. 10.1002/cpbi.5 27322403

[7] The UniProt Consortium UniProt: the universal protein knowledgebase in 2021. Nucleic Acids Res. 2021; 49:D480–D489. 33237286 10.1093/nar/gkaa1100 PMC7778908

[8] Türei D. , Korcsmáros T., Saez-Rodriguez J. OmniPath: guidelines and gateway for literature-curated signaling pathway resources. Nat. Methods. 2016; 13:966–967. 27898060 10.1038/nmeth.4077

[9] Knight J.D.R. , Samavarchi-Tehrani P., Tyers M., Gingras A.-C. Gene Information eXtension (GIX): effortless retrieval of gene product information on any website. Nat. Methods. 2019; 16:665–666. 31217594 10.1038/s41592-019-0477-9 PMC6669099

[10] Papatheodorou I. , Fonseca N.A., Keays M., Tang Y.A., Barrera E., Bazant W., Burke M., Füllgrabe A., Fuentes A.M.-P., George N. et al. Expression Atlas: gene and protein expression across multiple studies and organisms. Nucleic Acids Res. 2018; 46:D246–D251. 29165655 10.1093/nar/gkx1158 PMC5753389

[11] Schaab C. , Geiger T., Stoehr G., Cox J., Mann M. Analysis of high accuracy, quantitative proteomics data in the MaxQB database. Mol. Cell. Proteomics. 2012; 11:M111.014068. 10.1074/mcp.M111.014068 PMC3316731 22301388

[12] Wilkinson M.D. , Dumontier M., Aalbersberg Ij.J., Appleton G., Axton M., Baak A., Blomberg N., Boiten J.-W., da Silva Santos L.B., Bourne P.E. et al. The FAIR guiding principles for scientific data management and stewardship. Sci. Data. 2016; 3:160018. 26978244 10.1038/sdata.2016.18 PMC4792175

[13] Lamprecht A.-L. , Garcia L., Kuzak M., Martinez C., Arcila R., Martin Del Pico E., Dominguez Del Angel V., van de Sandt S., Ison J., Martinez P.A. et al. Towards FAIR principles for research software. DS. 2020; 3:37–59.

[14] Shraideh M. , Samaras P., Schreieck M., Krcmar H. Chandra Kruse L. , Seidel S., Hausvik G.I. A microservice-based reference architecture for digital platforms in the proteomics domain. The Next Wave of Sociotechnical Design. 2021; Cham Springer International Publishing 260–271.

[15] Gessulat S. , Schmidt T., Zolg D.P., Samaras P., Schnatbaum K., Zerweck J., Knaute T., Rechenberger J., Delanghe B., Huhmer A. et al. Prosit: proteome-wide prediction of peptide tandem mass spectra by deep learning. Nat. Methods. 2019; 16:509–518. 31133760 10.1038/s41592-019-0426-7

[16] Wilhelm M. , Zolg D.P., Graber M., Gessulat S., Schmidt T., Schnatbaum K., Schwencke-Westphal C., Seifert P., de Andrade Krätzig N., Zerweck J. et al. Deep learning boosts sensitivity of mass spectrometry-based immunopeptidomics. Nat. Commun. 2021; 12:3346. 34099720 10.1038/s41467-021-23713-9 PMC8184761

[17] Pezoa F. , Reutter J.L., Suarez F., Ugarte M., Vrgoč D. Foundations of JSON Schema. Proceedings of the 25th International Conference on World Wide Web, WWW ’16. International World Wide Web Conferences Steering Committee. 2016; Republic and Canton of Geneva, CHE 263–273.

[18] World Wide Web Consortium 2006; Extensible Markup Language (XML) 1.1.

[19] Bernhofer M. , Dallago C., Karl T., Satagopam V., Heinzinger M., Littmann M., Olenyi T., Qiu J., Schütze K., Yachdav G. et al. PredictProtein - predicting protein structure and function for 29 Years. Nucleic Acids Res. 2021; 49:W535–W540. 33999203 10.1093/nar/gkab354 PMC8265159

[20] Hecht M. , Bromberg Y., Rost B. Better prediction of functional effects for sequence variants. BMC Genomics. 2015; 16:S1. 10.1186/1471-2164-16-S8-S1 PMC4480835 26110438

[21] Letunic I. , Khedkar S., Bork P. SMART: recent updates, new developments and status in 2020. Nucleic Acids Res. 2021; 49:D458–D460. 33104802 10.1093/nar/gkaa937 PMC7778883

[22] Burley S.K. , Bhikadiya C., Bi C., Bittrich S., Chen L., Crichlow G.V., Christie C.H., Dalenberg K., Di Costanzo L., Duarte J.M. et al. RCSB Protein Data Bank: powerful new tools for exploring 3D structures of biological macromolecules for basic and applied research and education in fundamental biology, biomedicine, biotechnology, bioengineering and energy sciences. Nucleic Acids Res. 2021; 49:D437–D451. 33211854 10.1093/nar/gkaa1038 PMC7779003

[23] Schmidt T. , Samaras P., Dorfer V., Panse C., Kockmann T., Bichmann L., van Puyvelde B., Perez-Riverol Y., Deutsch E.W., Kuster B. et al. Universal Spectrum Explorer: A Standalone (Web-)Application for Cross-Resource spectrum comparison. J. Proteome Res. 2021; 20:3388–3394. 33970638 10.1021/acs.jproteome.1c00096

[24] Zolg D.P. , Wilhelm M., Schnatbaum K., Zerweck J., Knaute T., Delanghe B., Bailey D.J., Gessulat S., Ehrlich H.-C., Weininger M. et al. Building ProteomeTools based on a complete synthetic human proteome. Nat. Methods. 2017; 14:259–262. 28135259 10.1038/nmeth.4153 PMC5868332

[25] Verbruggen S. , Gessulat S., Gabriels R., Matsaroki A., Van de Voorde H., Kuster B., Degroeve S., Martens L., Van Criekinge W., Wilhelm M. et al. Spectral prediction features as a solution for the search space size problem in proteogenomics. Mol. Cell. Proteomics. 2021; 20:100076. 33823297 10.1016/j.mcpro.2021.100076 PMC8214147

[26] Martens L. , Vizcaíno J.A. A golden age for working with public proteomics data. Trends Biochem. Sci. 2017; 42:333–341. 28118949 10.1016/j.tibs.2017.01.001 PMC5414595

[27] Deutsch E.W. , Bandeira N., Sharma V., Perez-Riverol Y., Carver J.J., Kundu D.J., García-Seisdedos D., Jarnuczak A.F., Hewapathirana S., Pullman B.S. et al. The ProteomeXchange consortium in 2020: enabling ‘big data’ approaches in proteomics. Nucleic Acids Res. 2020; 48:D1145–D1152. 31686107 10.1093/nar/gkz984 PMC7145525

[28] Perez-Riverol Y. , Csordas A., Bai J., Bernal-Llinares M., Hewapathirana S., Kundu D.J., Inuganti A., Griss J., Mayer G., Eisenacher M. et al. The PRIDE database and related tools and resources in 2019: improving support for quantification data. Nucleic Acids Res. 2019; 47:D442–D450. 30395289 10.1093/nar/gky1106 PMC6323896

[29] Wang D. , Eraslan B., Wieland T., Hallström B., Hopf T., Zolg D.P., Zecha J., Asplund A., Li L., Meng C. et al. A deep proteome and transcriptome abundance atlas of 29 healthy human tissues. Mol. Syst. Biol. 2019; 15:e8503. 30777892 10.15252/msb.20188503 PMC6379049

[30] Savitski M.M. , Wilhelm M., Hahne H., Kuster B., Bantscheff M. A scalable approach for protein false discovery rate estimation in large proteomic data sets. Mol. Cell Proteomics. 2015; 14:2394–2404. 25987413 10.1074/mcp.M114.046995 PMC4563723

[31] Xu B. , Tian R., Wang X., Zhan S., Wang R., Guo Y., Ge W. Protein profile changes in the frontotemporal lobes in human severe traumatic brain injury. Brain Res. 2016; 1642:344–352. 27067185 10.1016/j.brainres.2016.04.008

[32] Beck K.L. , Weber D., Phinney B.S., Smilowitz J.T., Hinde K., Lönnerdal B., Korf I., Lemay D.G. Comparative proteomics of human and macaque milk reveals species-specific nutrition during postnatal development. J. Proteome Res. 2015; 14:2143–2157. 25757574 10.1021/pr501243m

[33] Zhang Y. , Li Q., Wu F., Zhou R., Qi Y., Su N., Chen L., Xu S., Jiang T., Zhang C. et al. Tissue-Based proteogenomics reveals that human testis endows plentiful missing proteins. J. Proteome Res. 2015; 14:3583–3594. 26282447 10.1021/acs.jproteome.5b00435

[34] Kollipara L. , Buchkremer S., Weis J., Brauers E., Hoss M., Rütten S., Caviedes P., Zahedi R.P., Roos A. Proteome profiling and ultrastructural characterization of the human RCMH cell line: Myoblastic properties and suitability for myopathological studies. J. Proteome Res. 2016; 15:945–955. 26781476 10.1021/acs.jproteome.5b00972

[35] Lawrence R.T. , Searle B.C., Llovet A., Villén J. Plug-and-play analysis of the human phosphoproteome by targeted high-resolution mass spectrometry. Nat. Methods. 2016; 13:431–434. 27018578 10.1038/nmeth.3811 PMC5915315

[36] Magdeldin S. , Hirao Y., Elguoshy A., Xu B., Zhang Y., Fujinaka H., Yamamoto K., Yates J.R., Yamamoto T. A proteomic glimpse into human ureter proteome. Proteomics. 2016; 16:80–84. 26442468 10.1002/pmic.201500214 PMC4737284

[37] Sharma K. , D’Souza R.C.J., Tyanova S., Schaab C., Wiśniewski J.R., Cox J., Mann M. Ultradeep human phosphoproteome reveals a distinct regulatory nature of tyr and Ser/Thr-Based signaling. Cell Rep. 2014; 8:1583–1594. 25159151 10.1016/j.celrep.2014.07.036

[38] Bhattacharjee M. , Balakrishnan L., Renuse S., Advani J., Goel R., Sathe G., Keshava Prasad T.S., Nair B., Jois R., Shankar S. et al. Synovial fluid proteome in rheumatoid arthritis. Clin. Proteome. 2016; 13:12. 10.1186/s12014-016-9113-1 PMC4893419 27274716

[39] Li L. , Wei Y., To C., Zhu C.-Q., Tong J., Pham N.-A., Taylor P., Ignatchenko V., Ignatchenko A., Zhang W. et al. Integrated omic analysis of lung cancer reveals metabolism proteome signatures with prognostic impact. Nat. Commun. 2014; 5:5469. 25429762 10.1038/ncomms6469

[40] Slebos R.J.C. , Wang X., Wang X., Wang X., Zhang B., Tabb D.L., Liebler D.C. Proteomic analysis of colon and rectal carcinoma using standard and customized databases. Sci. Data. 2015; 2:150022. 26110064 10.1038/sdata.2015.22 PMC4477697

[41] Tan H. , Wu Z., Wang H., Bai B., Li Y., Wang X., Zhai B., Beach T.G., Peng J. Refined phosphopeptide enrichment by phosphate additive and the analysis of human brain phosphoproteome. Proteomics. 2015; 15:500–507. 25307156 10.1002/pmic.201400171 PMC4598062

[42] Xu B. , Gao Y., Zhan S., Xiong F., Qiu W., Qian X., Wang T., Wang N., Zhang D., Yang Q. et al. Quantitative protein profiling of hippocampus during human aging. Neurobiol. Aging. 2016; 39:46–56. 26923401 10.1016/j.neurobiolaging.2015.11.029

[43] Adachi J. , Kishida M., Watanabe S., Hashimoto Y., Fukamizu K., Tomonaga T. Proteome-wide discovery of unknown ATP-binding proteins and kinase inhibitor target proteins using an ATP probe. J. Proteome Res. 2014; 13:5461–5470. 25230287 10.1021/pr500845u

[44] Jumeau F. , Com E., Lane L., Duek P., Lagarrigue M., Lavigne R., Guillot L., Rondel K., Gateau A., Melaine N. et al. Human spermatozoa as a model for detecting missing proteins in the context of the chromosome-centric human proteome project. J. Proteome Res. 2015; 14:3606–3620. 26168773 10.1021/acs.jproteome.5b00170

[45] Vandenbrouck Y. , Lane L., Carapito C., Duek P., Rondel K., Bruley C., Macron C., Gonzalez de Peredo A., Couté Y., Chaoui K. et al. Looking for missing proteins in the proteome of human spermatozoa: An update. J. Proteome Res. 2016; 15:3998–4019. 27444420 10.1021/acs.jproteome.6b00400

[46] Kroksveen A.C. , Guldbrandsen A., Vaudel M., Lereim R.R., Barsnes H., Myhr K.-M., Torkildsen Ø., Berven F.S. In-Depth cerebrospinal fluid quantitative proteome and deglycoproteome analysis: Presenting a comprehensive picture of pathways and processes affected by multiple sclerosis. J. Proteome Res. 2017; 16:179–194. 27728768 10.1021/acs.jproteome.6b00659

[47] Giansanti P. , Aye T.T., van den Toorn H., Peng M., van Breukelen B., Heck A.J.R. An augmented multiple-protease-based human phosphopeptide atlas. Cell Rep. 2015; 11:1834–1843. 26074081 10.1016/j.celrep.2015.05.029

[48] Piersma S.R. , Knol J.C., de Reus I., Labots M., Sampadi B.K., Pham T.V., Ishihama Y., Verheul H.M.W., Jimenez C.R. Feasibility of label-free phosphoproteomics and application to base-line signaling of colorectal cancer cell lines. J. Proteomics. 2015; 127:247–258. 25841592 10.1016/j.jprot.2015.03.019

[49] Hao P. , Ren Y., Pasterkamp G., Moll F.L., de Kleijn D.P.V., Sze S.K. Deep proteomic profiling of human carotid atherosclerotic plaques using multidimensional LC-MS/MS. Proteome Clin. Appl. 2014; 8:631–635. 10.1002/prca.201400007 24828403

[50] Billing A.M. , Ben Hamidane H., Dib S.S., Cotton R.J., Bhagwat A.M., Kumar P., Hayat S., Yousri N.A., Goswami N., Suhre K. et al. Comprehensive transcriptomic and proteomic characterization of human mesenchymal stem cells reveals source specific cellular markers. Sci. Rep. 2016; 6:21507. 26857143 10.1038/srep21507 PMC4746666

[51] Robertson J. , Jacquemet G., Byron A., Jones M.C., Warwood S., Selley J.N., Knight D., Humphries J.D., Humphries M.J. Defining the phospho-adhesome through the phosphoproteomic analysis of integrin signalling. Nat. Commun. 2015; 6:6265. 25677187 10.1038/ncomms7265 PMC4338609

[52] Kim M.-S. , Zhong Y., Yachida S., Rajeshkumar N.V., Abel M.L., Marimuthu A., Mudgal K., Hruban R.H., Poling J.S., Tyner J.W. et al. Heterogeneity of pancreatic cancer metastases in a single patient revealed by quantitative proteomics. Mol. Cell. Proteomics. 2014; 13:2803–2811. 24895378 10.1074/mcp.M114.038547 PMC4223473

[53] Murthy K.R. , Rajagopalan P., Pinto S.M., Advani J., Murthy P.R., Goel R., Subbannayya Y., Balakrishnan L., Dash M., Anil A.K. et al. Proteomics of human aqueous humor. OMICS. 2015; 19:283–293. 25933257 10.1089/omi.2015.0029

[54] Osinalde N. , Sanchez-Quiles V., Akimov V., Guerra B., Blagoev B., Kratchmarova I. Simultaneous dissection and comparison of IL-2 and IL-15 signaling pathways by global quantitative phosphoproteomics. Proteomics. 2015; 15:520–531. 25142963 10.1002/pmic.201400194

[55] Naboulsi W. , Megger D.A., Bracht T., Kohl M., Turewicz M., Eisenacher M., Voss D.M., Schlaak J.F., Hoffmann A.-C., Weber F. et al. Quantitative tissue proteomics analysis reveals versican as potential biomarker for early-stage hepatocellular carcinoma. J. Proteome Res. 2016; 15:38–47. 26626371 10.1021/acs.jproteome.5b00420

[56] Bracht T. , Schweinsberg V., Trippler M., Kohl M., Ahrens M., Padden J., Naboulsi W., Barkovits K., Megger D.A., Eisenacher M. et al. Analysis of disease-associated protein expression using quantitative proteomics—fibulin-5 is expressed in association with hepatic fibrosis. J. Proteome Res. 2015; 14:2278–2286. 25807371 10.1021/acs.jproteome.5b00053

[57] Wu X. , Zahari M.S., Ma B., Liu R., Renuse S., Sahasrabuddhe N.A., Chen L., Chaerkady R., Kim M.-S., Zhong J. et al. Global phosphotyrosine survey in triple-negative breast cancer reveals activation of multiple tyrosine kinase signaling pathways. Oncotarget. 2015; 6:29143–29160. 26356563 10.18632/oncotarget.5020 PMC4745717

[58] Tyanova S. , Albrechtsen R., Kronqvist P., Cox J., Mann M., Geiger T. Proteomic maps of breast cancer subtypes. Nat. Commun. 2016; 7:10259. 26725330 10.1038/ncomms10259 PMC4725767

[59] Svinkina T. , Gu H., Silva J.C., Mertins P., Qiao J., Fereshetian S., Jaffe J.D., Kuhn E., Udeshi N.D., Carr S.A. Deep, quantitative coverage of the lysine acetylome using novel anti-acetyl-lysine antibodies and an optimized proteomic workflow. Mol. Cell. Proteomics. 2015; 14:2429–2440. 25953088 10.1074/mcp.O114.047555 PMC4563726

[60] Huttlin E.L. , Jedrychowski M.P., Elias J.E., Goswami T., Rad R., Beausoleil S.A., Villén J., Haas W., Sowa M.E., Gygi S.P. A tissue-specific atlas of mouse protein phosphorylation and expression. Cell. 2010; 143:1174–1189. 21183079 10.1016/j.cell.2010.12.001 PMC3035969

[61] Kole K. , Lindeboom R.G.H., Baltissen M.P.A., Jansen P.W.T.C., Vermeulen M., Tiesinga P., Celikel T. Proteomic landscape of the primary somatosensory cortex upon sensory deprivation. Gigascience. 2017; 6:1–10. 10.1093/gigascience/gix082 PMC5632293 29020746

[62] Sharma K. , Schmitt S., Bergner C.G., Tyanova S., Kannaiyan N., Manrique-Hoyos N., Kongi K., Cantuti L., Hanisch U.-K., Philips M.-A. et al. Cell type- and brain region-resolved mouse brain proteome. Nat. Neurosci. 2015; 18:1819–1831. 26523646 10.1038/nn.4160 PMC7116867

[63] Branca R.M.M. , Orre L.M., Johansson H.J., Granholm V., Huss M., Pérez-Bercoff Å., Forshed J., Käll L., Lehtiö J. HiRIEF LC-MS enables deep proteome coverage and unbiased proteogenomics. Nat. Methods. 2014; 11:59–62. 24240322 10.1038/nmeth.2732

[64] Kähne T. , Richter S., Kolodziej A., Smalla K.-H., Pielot R., Engler A., Ohl F.W., Dieterich D.C., Seidenbecher C., Tischmeyer W. et al. Proteome rearrangements after auditory learning: high-resolution profiling of synapse-enriched protein fractions from mouse brain. J. Neurochem. 2016; 138:124–138. 27062398 10.1111/jnc.13636 PMC5089584

[65] Xu B. , Xiong F., Tian R., Zhan S., Gao Y., Qiu W., Wang R., Ge W., Ma C. Temporal lobe in human aging: a quantitative protein profiling study of samples from Chinese Human Brain Bank. Exp. Gerontol. 2016; 73:31–41. 26631761 10.1016/j.exger.2015.11.016

[66] Bettica P. , Petrini S., D’Oria V., D’Amico A., Catteruccia M., Pane M., Sivo S., Magri F., Brajkovic S., Messina S. et al. Histological effects of givinostat in boys with Duchenne muscular dystrophy. Neuromuscul. Disord. 2016; 26:643–649. 27566866 10.1016/j.nmd.2016.07.002

[67] Lechner S. , Malgapo M., Grätz C., Baron A., Rüther P.L., Nadal S., Stumpf C., Loos C., Ku X., Prokofeva P. et al. Target deconvolution of HDAC pharmacopoeia highlights MBLAC2 as common off-target. 2021; Research Square doi:2 July 2021, preprint: not peer reviewed 10.21203/rs.3.rs-646613/v1. PMC9339481 35484434

